# Mesenchymal Stem Cell Transplantation Ameliorates Ara-C-Induced Motor Deficits in a Mouse Model of Cerebellar Ataxia

**DOI:** 10.3390/jcm12051756

**Published:** 2023-02-22

**Authors:** Narae Park, Chanchal Sharma, Un Ju Jung, Sehwan Kim, Youngpyo Nam, Kyung-Suk Kim, Kyoungho Suk, Ho-Won Lee, Sang Ryong Kim

**Affiliations:** 1 School of Life Sciences, Kyungpook National University, Daegu 41566, Republic of Korea; 2 BK21 FOUR KNU Creative BioResearch Group, Kyungpook National University, Daegu 41566, Republic of Korea; 3 Department of Food Science and Nutrition, Pukyong National University, Busan 48513, Republic of Korea; 4 Brain Science and Engineering Institute, Kyungpook National University, Daegu 41404, Republic of Korea; 5 Bioengineering Institute, Corestem Inc., Seoul 13486, Republic of Korea; 6 Department of Pharmacology, School of Medicine, Kyungpook National University, Daegu 41944, Republic of Korea; 7 Department of Neurology, Kyungpook National University Chilgok Hospital, Daegu 41404, Republic of Korea

**Keywords:** human mesenchymal stem cells, cytosine arabinoside, cerebellar ataxia, motor behavior, neurotrophic factor

## Abstract

This study investigated the therapeutic effects of transplanting human mesenchymal stem cells (hMSCs) into wild-type mice that were intraperitoneally administered cytosine arabinoside (Ara-C) to develop cerebellar ataxia (CA) during the first three postnatal days. hMSCs were intrathecally injected into 10-week-old mice once or thrice at 4-week intervals. Compared to the nontreated mice, the hMSC-treated mice showed improved motor and balance coordination, as measured using the rotarod, open-field, and ataxic scoring assessments, and increased protein levels in Purkinje and cerebellar granule cells, as measured using calbindin and NeuN protein markers. Multiple hMSC injections preserved Ara-C-induced cerebellar neuronal loss and improved cerebellar weight. Furthermore, the hMSC implantation significantly elevated the levels of neurotrophic factors, including brain-derived and glial cell line-derived neurotrophic factors, and suppressed TNF-α-, IL-1β-, and iNOS-mediated proinflammatory responses. Collectively, our results demonstrate that hMSCs exhibit therapeutic potential for Ara-C-induced CA by protecting neurons through the stimulation of neurotrophic factors and inhibition of cerebellar inflammatory responses, which can improve motor behavior and alleviate ataxia-related neuropathology. In summary, this study suggests that hMSC administration, particularly multiple treatments, can effectively treat ataxia-related symptoms with cerebellar toxicity.

## 1. Introduction

Cerebellar ataxia (CA) is a group of heterogeneous disorders caused by cerebellar dysfunction, typically manifesting symptoms of unsteady gait, abnormal eye movements, and slurred speech, among others; CA results from a genetic deficit or spontaneous causes [1,2]. Although several studies have attempted to investigate physiological, molecular, and biochemical alterations in patients with CA and various model systems to identify new therapeutic targets, most patients with CA have an unidentified etiology. Thus, no complete treatment for CA is available and only symptom mitigation is possible [3,4].

Mesenchymal stem cells (MSCs) are fibroblast-like, plate-adhering cells with the capacity for self-renewal and multidifferentiation. They are promising therapeutic agents for various neurological diseases, including Parkinson’s disease and Alzheimer’s disease [5,6,7,8,9]. Compared to embryonic stem cells and other tissue-specific stem cells (e.g., hematological and neural stem cells), human MSCs (hMSCs) have several advantages, including easy availability and ability to be isolated from various organs, such as fetal lung and liver, as well as tissues, such as bone marrow, umbilical cord blood, trabecular bone, synovial membrane, and adipose tissue [10,11,12]. They are also associated with fewer ethical concerns and are less likely to cause immunogenicity, thus opening avenues for their application in human clinical trials. In addition, they have strong proliferation abilities in vitro while retaining a multipotent, undifferentiated state [13]. They also exert neurotrophic and immunomodulatory effects via the diffusion of several chemicals, such as cytokines, chemokines, growth factors, and neurotrophic factors, and therapeutic benefits via differentiation into neural cells [14,15]. Additionally, preclinical studies in animals have suggested that hMSC implantation restores motor function and preserves cerebellar neurons in various CA mouse models [10,12,16,17,18,19,20,21,22]. Thus, hMSCs are an ideal candidate for MSC-based therapies.

We previously revealed that hMSC treatment inhibited neuroinflammatory symptoms in a lipopolysaccharide (LPS)-induced CA mouse model [19], modulated microglial M2 polarization, and inhibited apoptosis in the LPS-induced CA mice [20]. Other reports have also suggested that hMSC administration attenuates motor dysfunction and neurodegeneration in mouse models of spinocerebellar ataxia (SCA) type 1, 2, and 3 [8,17,18,23]. Furthermore, umbilical cord-derived hMSCs can reportedly inhibit mutant ataxin-3 toxicity via modulation of the IGF-1/heat-shock protein-70 pathway [17]. However, the short lifetime of hMSCs is a concern [16,21,22]. Thus, we examined the effects of single or multiple administration of hMSCs in a cytosine arabinoside (Ara-C)-treated mouse model of CA, which exhibits CA-like symptoms, including incoordination, motor imbalance, and neuronal damage in the cerebellum [24,25,26].

## 2. Material and Methods

### 2.1. Animals and Disease Model

**Figure 1:**
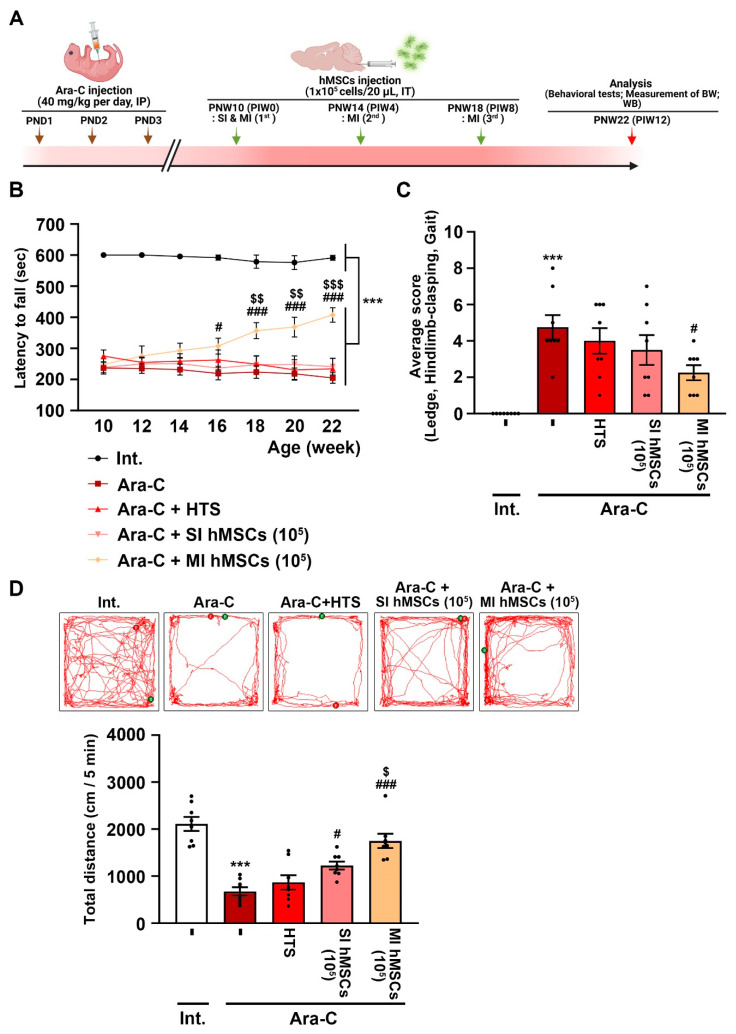
Beneficial effects of hMSCs on behavioral impairments in Ara-C-induced CA mice. (**A**) Experimental schematic of hMSC treatment in an Ara-C-induced CA mouse model. The figure was obtained using BioRender.com (Agreement number: FP24TP0Q0J). To establish a chemically induced animal model of CA, Ara-C (40 mg/kg/day) was intraperitoneally injected with a 0.3 mL syringe daily on postnatal days 1–3. The hMSC transplantation was performed once (single treatment) or thrice (multiple treatments) at 4-week intervals in 10-week-old Ara-C-induced CA mice. (**B**) A behavioral test for motor coordination impairment was performed using the rotarod test in the Ara-C-induced CA mice treated with hMSCs from 10 to 22 weeks of age at 2-week intervals. *** *p* < 0.001 vs. intact mice; ^#^ *p* < 0.05, ^###^ *p* < 0.001 vs. Ara-C-induced CA mice; ^$$^ *p* < 0.01, ^$$$^ *p* < 0.001 vs. single hMSC treatment in Ara-C-induced CA mice (one-way analysis of variance (ANOVA) with Tukey’s post hoc analysis; *n* = 8 for each group). (**C**) A simple composite phenotype scoring system in the Ara-C-induced CA mice at 12 weeks after hMSC treatment. *** *p* < 0.001 vs. intact mice; ^#^ *p* < 0.05 vs. Ara-C-induced CA mice (one-way ANOVA with Tukey’s post hoc analysis; *n* = 8 for each group). (**D**) A behavioral test for locomotor activity was performed using the open-field test in the Ara-C-induced CA mice at 12 weeks after hMSC treatment. *** *p* < 0.001 vs. intact mice; ^#^ *p* < 0.05, ^###^ *p* < 0.001 vs. Ara-C-induced CA mice; ^$^ *p* < 0.05 vs. single hMSC treatment in Ara-C-induced CA mice (one-way ANOVA with Tukey’s post hoc analysis; *n* = 8 for each group). Int., intact; Ara-C, cytosine arabinoside; HTS, hypothermosol; hMSCs, human mesenchymal stem cells; SI, single injection; MI, multiple injection; PND, postnatal day; PNW, postnatal week; PIW, post implantation week (hMSC); IP, intraperitoneal injection; IT, intrathecal injection; CW, cerebellar weight; WB, Western blot.

To establish a chemically induced animal model of CA, cytosine β-D-arabinofuranoside hydrochloride (Ara-C; 40 mg/kg/day) purchased from Sigma-Aldrich was dissolved in normal saline (0.9% NaCl), and the solution was intraperitoneally injected into C57BL/6 mice (Daehan Biolink Co., Ltd., Eumseong, Republic of Korea) during the first 3 postnatal days (Figure 1A). The mice were housed in a controlled environment (23 °C + 2 °C; 50–60% humidity; 12 h light/dark cycle) with ad libitum access to food and water. All experimental animal procedures were conducted according to the approved animal protocols and guidelines of the Animal Care Committee of Kyungpook National University (No. KNU 2019-0002 and 2022-0018). The experimental animals were treated humanely, and the number of animals involved in the experiment was minimized.

### 2.2. hMSC Implantation

hMSCs were prepared in accordance with the protocols approved by the Institutional Review Board of Chilgok Kyungpook National University Hospital (IRB: 2017-11-015-007) [20]. Briefly, the bone marrow samples were obtained from eight healthy donors. The cells were seeded at a density of 11.5 × 10^7^ cells/cm^2^ and cultured in an CSBM-A06 medium (Corestem Inc., Seoul, Republic of Korea) supplemented with 10% fetal bovine serum (Life Technologies-Thermo Fisher Scientific, Waltham, MA USA), 2.5 mM of L-alanyl-L-glutamine (Merck Biochrom AG, Berlin, Germany), and 1% penicillin–streptomycin (Merck Biochrom AG, Berlin, Germany). Nonadherent and dead cells were washed out, and the medium was replenished twice a week. Passage 0 (P0) cells were defined as adherent cells that attained up to 70–80% confluence. At P5, the cells were analyzed for the expression of cell surface markers using flow cytometry. hMSCs were identified as CD29/CD44/CD73/CD105-positive and CD34/CD45-negative cells.

For hMSC implantation, the mice were randomly divided into five experimental groups: nontreated intact mice (Group 1), Ara-C-induced CA mice (Group 2), Ara-C-induced CA mice treated with hypothermosol (HTS) (Group 3), single-hMSC-administered Ara-C-induced CA mice (Group 4), and multiple-hMSC-administered Ara-C-induced CA mice (Group 5). The mice were also treated with HTS, an optimized preservation medium used to maintain stem cells at low temperatures and mitigate temperature-induced molecular cell stress responses, which was used as a control for the hMSC administrations. The mice’s head was positioned at an angle of approximately 90° with their body in the stereotaxic frame. Using a Hamilton syringe (25 µL, 30-gauge) attached to a syringe pump, hMSCs (1 × 10^5^ cells/20 µL) were injected into the cisterna magna after being exposed to the underlying dura mater (2 µL/min). To prevent the reflux of cerebrospinal fluid, we removed the syringe after 10 min and sealed the incision with a silk suture.

### 2.3. Behavioral Tests

The rotarod test was conducted 1 day before hMSC transplantation (postnatal week [PNW] 10) and in the next 12 weeks (22-week-old mice [PNW 12]) at 2-week intervals, as described previously [27]. The experimental mice were carefully placed on a rotating apparatus (3 cm rod diameter). The rotational speed was linearly increased from 4 to 40 rpm over 5 min and was maintained at this speed for the next 5 min. The total time that the mice remained on the apparatus was recorded. Each mouse received a 10 min rest interval between trials to avoid muscle fatigue.

The simple composite phenotype scoring system comprised a ledge test, a hindlimb clasping test, and a gait test, and it was performed in 22-week-old mice at 12 weeks after hMSC transplantation (PNW 22), as described previously [28,29]. All tests were scored on a scale of 0–3, where 0 indicates the absence of the relevant phenotype and 3 indicates the most severe symptoms. These test scores were further combined to obtain a composite phenotype score of 0–9. Each test was conducted thrice. The ledge test [28,29] directly measures coordination, which is impaired by CA. A score of 0 was assigned when the mice did not lose balance and walked along the ledge; a score of 1 was assigned when the mice walked in an asymmetrical posture along the ledge; a score of 2 was assigned when the hind legs were not used by the mice; and a score of 3 was assigned when the mice fell off the ledge. The hindlimb clasping test [28,29] is an indicator of CA progression. A score of 0 was assigned when the hindlimbs were consistently splayed outward, away from the abdomen. A score of 1 or 2 was assigned when the mice retracted one or both hindlimbs toward the abdomen for >50% of the total measurement duration, respectively. Additionally, a score of 3 was assigned when the mice’s hindlimbs were entirely retracted and touched their abdomen for >50% of the total measurement duration. The gait test [28,29] was performed to evaluate coordination and muscle function. After placing the mice on a flat surface, they were observed while walking. A score of 0 was assigned when the mice moved normally with their body weight supported on all limbs and without their abdomen touching the ground; a score of 1 was assigned when the mice showed a tremor or seemed to be limping while walking; a score of 2 was assigned when the mice showed a severe tremor, severe limp, lowered pelvis, or duck step during locomotion; and a score of 3 was assigned when the mice showed difficulty moving forward and dragged their abdomen along the ground.

The open-field test was used to measure the locomotor activity once after 12 weeks of hMSC transplantation in 22-week-old mice with some modifications [30]. The behavior of the mice was recorded for 6 min using a video recording system after they were individually placed in the corner of the arena of a white acrylic box (40 × 40 × 40 cm). The recorded behavior was analyzed using the SMART 3.0 video-tracking software (Panlab-SMART). The arena was wiped with 70% ethanol between tests. The tests were performed in an environment with low luminous intensity to minimize stress.

### 2.4. Assessment of Cerebellar Weight

Cerebellar weight was measured using a previously described method with some modifications [31]. All mouse brains from each experimental group were dissected from their skull and were sectioned from their spinal cord and cranial nerves. A small knife was inserted parallel to the dorsal hindbrain to excise the peduncles and separate the cerebellum from the brain. The mouse cerebellar weight was measured up to approximately 0.1 mg. The entire process was conducted at room temperature.

### 2.5. Western Blot Analysis

Western blot was performed based on previously described methods with some modifications [20]. Briefly, the cerebellar vermis of the mice was homogenized in a lysis buffer (2% SDS, 10% glycerol, 58 mM Tris-HCl; pH 6.8) supplemented with a phosphatase inhibitor cocktail (1:100, Cell Signaling Technology, Danvers, MA, USA) and a protease inhibitor cocktail (1:100, Cell Signaling Technology, Danvers, MA, USA). The lysate was further centrifuged for 15 min at 14,000× *g* and 4 °C, and the supernatant was transferred to a fresh tube. The protein concentration was measured using a Bicinchoninic Acid Assay Kit (Bio-Rad Laboratories, Hercules, CA, USA). An equal amount of protein was electrophoresed using a sodium dodecyl sulfate/polyacrylamide gel (Bio-Rad Laboratories, Hercules, CA, USA) and transferred to polyvinylidene difluoride membranes (Millipore) via an electrophoretic transfer system (Bio-Rad Laboratories, Hercules, California). The membranes were incubated with the following primary antibodies at 4 °C for 48 h: mouse anti-calbindin (1:2000; Sigma-Aldrich, St. Louis, MO, USA), mouse anti-NeuN (1:1000; Merck Millipore, MA, USA), mouse anti-glial cell line-derived neurotrophic factor (GDNF; 1:1000; R&D Systems, Minneapolis, MN, USA), rabbit anti-brain-derived neurotrophic factor (BDNF; 1:1000; Santa Cruz Biotechnology, Dallas, TX, USA), rabbit anti-IL-1β (1:1,000; Santa Cruz Biotechnology, Dallas, TX, USA), rabbit anti-TNF-α (1:1000; Santa Cruz Biotechnology, Dallas, TX, USA), rabbit anti-iNOS (1:1000; Abcam, Waltham, MA, USA), and mouse anti-β-actin (1:2000; Santa Cruz Biotechnology, Dallas, TX, USA). The membranes were subsequently washed and incubated with horse radish peroxidase-conjugated secondary antibodies, including anti-mouse IgG (1:4000; Invitrogen-Fisher Scientific, Waltham, MA, USA) and anti-rabbit IgG (1:4000; Invitrogen-Fisher Scientific, Waltham, MA, USA), at room temperature for 1 h, after which the membranes were developed using enhanced chemiluminescence Western blot detection reagents (GE Healthcare Life Sciences, Chicago, IL, USA). The resultant signal was analyzed using a LAS-500 image analyzer (GE Healthcare Life Sciences, Chicago, IL, USA). The band density of the target protein was measured using the Multi Gauge version 3.0 (Fuji Film) and was normalized to β-actin for each sample.

### 2.6. Statistical Analysis

All values are expressed as mean ± standard error of means (Mean ± SEM). Differences between groups were assessed using a one-way analysis of variance, followed by Tukey’s post hoc analysis. All statistical analyses were conducted using GraphPad Prism (version 8.30; GraphPad Software, San Diego, CA, USA).

## 3. Results

### 3.1. Effects of hMSC Treatment on Impaired Behaviors of Ara-C-Induced CA Mice

To investigate the beneficial effects of hMSC (1 × 10^5^ cells) treatment in the Ara-C (40 mg/kg/day)-induced CA mice, we evaluated motor coordination and balance using the rotarod test every 2 weeks for the next 12 weeks after hMSC treatment (PNW 22), starting at 10 weeks of age (PNW 10) (Figure 1A). The Ara-C mice were subsequently treated with HTS, which is an optimized preservation medium used to maintain stem cells at low temperatures and mitigate temperature-induced molecular cell stress responses that occur during the chilling and thawing of cells. Moreover, we conducted experiments to compare single and multiple injections of hMSCs. In the rotarod test, compared to the nontreated intact mice (591.25 ± 8.24 s), the Ara-C-induced CA mice showed significant declined retention time (204.72 ± 17.0 s) on the rotating rod (Figure 1B; *** *p* < 0.001 vs. nontreated intact mice; *n* = 8). However, in the Ara-C-induced CA mice, multiple hMSC treatments (407.53 ± 23.15 s) significantly recovered the retention time for 22-week-old mice (PNW 22) compared to no treatment (204.72 ± 17.0) or single hMSC treatment (241.21 ± 27.2 s) (Figure 1B; ^###^ *p* < 0.001 vs. Ara-C-induced CA mice; ^$$$^ *p* < 0.001 vs. single hMSC treatment in Ara-C-induced CA mice; *n* = 8). The composite ataxia phenotype tests performed in 22-week-old mice (PNW 22) demonstrated that multiple hMSC treatments (2.25 ± 0.41) significantly mitigated the Ara-C-induced motor impairment (ledge and gait tests) and abnormal hindlimb clasping compared to no treatment in the Ara-C-induced CA mice (4.75 ± 0.67) (Figure 1C; *** *p* < 0.001 vs. nontreated intact mice; ^#^ *p* < 0.05 vs. Ara-C-induced CA mice; *n* = 8). Consistent with the rotarod test and phenotype scoring analysis, the open-field test at PNW 22 for assessing locomotor activity indicated that multiple hMSC treatments (1750 ± 150.4 cm) significantly reduced Ara-C-induced impaired locomotor activity (671.1 ± 90.19 cm) (Figure 1D; *** *p* < 0.001 vs. nontreated intact mice; ^###^ *p* < 0.001 vs. Ara-C-induced CA mice; ^$^ *p* < 0.05 vs. single hMSC treatment in Ara-C-induced CA mice; *n* = 8).

### 3.2. Effects of hMSC Treatment on Cell Damage in the Cerebellum of Ara-C-Induced CA Mice

**Figure 2:**
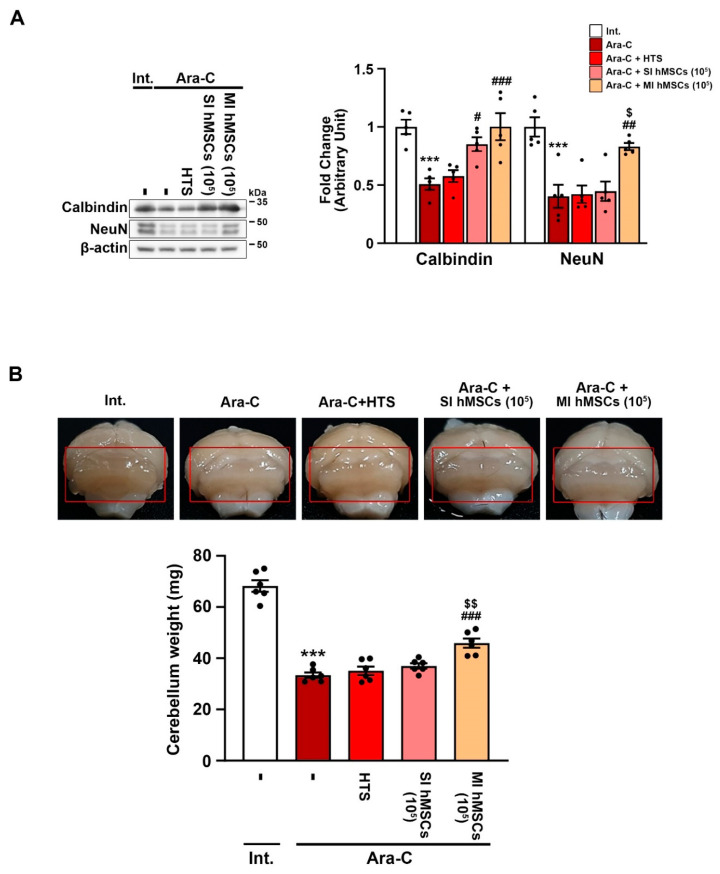
Treatment with hMSCs protects neurons in the cerebellum of Ara-C-induced CA mice. (**A**) Western blot analysis of calbindin (Purkinje cell marker) and NeuN (granule cell marker) in the cerebellum at 12 weeks after hMSC treatment. *** *p* < 0.001 vs. intact mice; ^#^ *p* < 0.05, ^##^ *p* < 0.01, ^###^ *p* < 0.001 vs. Ara-C-induced CA mice; ^$^ *p* < 0.05 vs. single hMSC treatment in Ara-C-induced CA mice (one-way analysis of variance [ANOVA] with Tukey’s post hoc analysis; *n* = 5 for each group). (**B**) The measurement of cerebellum weight at 12 weeks after hMSC treatment. *** *p* < 0.001 vs. intact mice; ^###^ *p* < 0.001 vs. Ara-C-induced CA mice; ^$$^ *p* < 0.01 vs. single hMSC treatment in Ara-C-induced CA mice (one-way ANOVA with Tukey’s post hoc analysis; *n* = 6 for each group). Int., intact; Ara-C, cytosine arabinoside; HTS, hypothermosol; hMSCs, human mesenchymal stem cells; SI, single injection; MI, multiple injection; NeuN, neuronal nuclei.

To investigate the effects of hMSCs on cerebellar cell damage caused by Ara-C-induced neurotoxicity, we measured the levels of calbindin (a marker of Purkinje cell) and NeuN (a marker of granule cell) in the cerebellum of the intact mice, the Ara-C mice, and the hMSC-treated Ara-C mice using Western blot analysis at PNW22 (Figure 2A). The Western blot analysis revealed a significant decrease in the protein levels of calbindin in the cerebellum of the Ara-C-induced CA mice (0.508 ± 0.049) compared to the intact mice (1 ± 0.061) (Figure 2A; *** *p* < 0.001 vs. nontreated intact mice; *n* = 5). The protein levels of calbindin were preserved in the cerebellum of the Ara-C-induced CA mice in the hMSC treatment groups, including the single (0.851 ± 0.059) and multiple (1.002 ± 0.116) injection groups (Figure 2A; ^###^ *p* < 0.001 vs. Ara-C-induced CA mice; *n* = 5). However, the protein levels of NeuN were only preserved via multiple hMSC treatments (0.831 ± 0.03) in the cerebellum of the CA mice (Figure 2A; *** *p* < 0.001 vs. nontreated intact mice; ^##^ *p* < 0.01 vs. Ara-C-induced CA mice; ^$^ *p* < 0.05 vs. single hMSC treatment in Ara-C-induced CA mice; *n* = 5).

Next, to evaluate whether the hMSC treatment protected from cerebellar atrophy, we measured the cerebellar weight in the animal models of CA (Figure 2B). The cerebellar weight of the Ara-C-induced CA mice (33.37 ± 1.09 mg) was significantly lower than that of the intact mice (68.23 ± 2.25 mg) (Figure 2B; *** *p* < 0.001 vs. nontreated intact mice; *n* = 6). However, multiple hMSC treatments attenuated Ara-C-induced loss of cerebellar weight (45.9 ± 1.8 mg) (Figure 2B; ^###^ *p* < 0.001 vs. Ara-C-induced CA mice; ^$$^ *p* < 0.01 vs. single hMSC treatment in Ara-C-induced CA mice; *n* = 6). Thus, multiple hMSC treatments may protect cerebellar neurons from Ara-C-induced neurotoxicity.

### 3.3. Increased Levels of Neurotrophic Factors and Anti-Inflammatory Effects via hMSC Treatment in the Cerebellum of CA Mice

**Figure 3:**
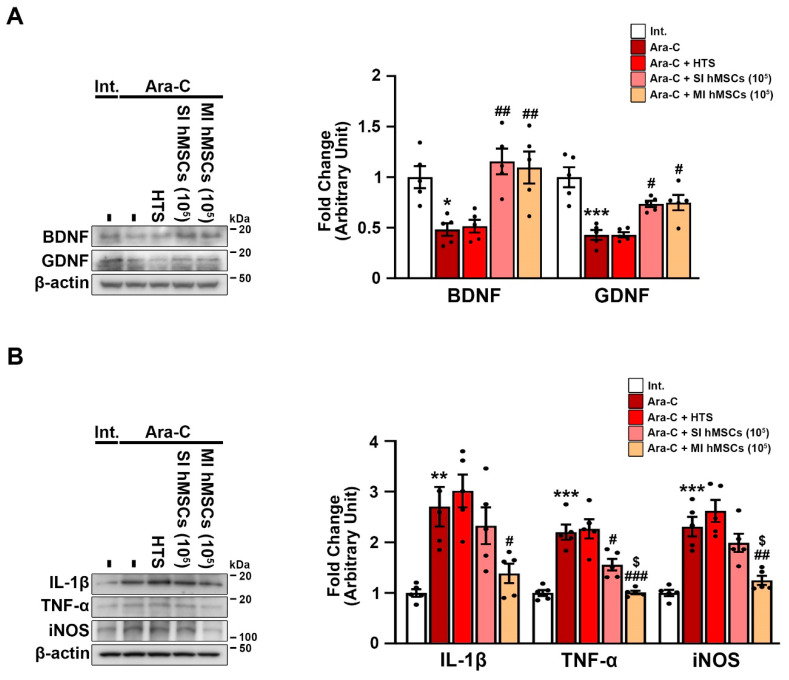
Treatment with hMSCs increases the protein levels of neurotrophic factors and decreases the protein levels of neurotoxic inflammatory molecules in the cerebellum of the CA mouse model. (**A**) Western blot analysis of GDNF and BDNF in the cerebellum at 12 weeks after hMSC treatment. * *p* < 0.05, *** *p* < 0.001 vs. intact mice; ^#^ *p* < 0.05, ^##^ *p* < 0.01 vs. Ara-C-induced CA mice (one-way analysis of variance [ANOVA] with Tukey’s post hoc analysis; *n* = 5 for each group). (**B**) Western blot analysis of IL-1β, TNF-α, and iNOS in the cerebellum at 12 weeks after hMSC treatment. ** *p* < 0.01, *** *p* < 0.001 vs. intact mice; ^#^ *p* < 0.05, ^##^ *p* < 0.01, ^###^ *p* < 0.001 vs. Ara-C-induced CA mice; ^$^ *p* < 0.05 vs. single hMSC treatment in Ara-C-induced CA mice (one-way ANOVA with Tukey’ post hoc analysis; *n* = 5 for each group). Int., intact; Ara-C, cytosine arabinoside; HTS, hypothermosol; hMSCs, human mesenchymal stem cells; SI, single injection; MI, multiple injection; BDNF, brain-derived neurotrophic factor; GDNF, glial-derived neurotrophic factor; IL-1β, interleukin-1β; TNF-α, tumor necrosis factor-α; iNOS, inducible nitric oxide synthase.

In the brains of individuals with degenerative diseases, hMSCs have beneficial effects on the production of neurotrophic factors [32] and suppression of inflammation [20]. BDNF and GDNF are the most active neurotrophic factors and reflect a compensatory mechanism against early neurodegeneration that may be related to inflammation. To determine whether the decrease in neurotoxicity induced by the hMSC treatment in the Ara-C-induced CA mice was associated with neurotrophic factors, we measured the protein levels of BDNF and GDNF in the cerebellum of the adult mice after 12 weeks of hMSC administration (PND 22) (Figure 3A). The Western blot results revealed a significant decrease in the protein levels of BDNF (0.483 ± 0.062) and GDNF (0.429 ± 0.048) in the cerebellum of the Ara-C-induced CA mice compared to those in the cerebellum of the nontreated intact mice (1 ± 0.108; 1 ± 0.099) (Figure 3A; *** *p* < 0.001 vs. nontreated intact mice; *n* = 5). The Ara-C-induced CA mice in the single (1.156 ± 0.127; 0.736 ± 0.032) and multiple hMSC injection groups (1.096 ± 0.159; 0.748 ± 0.075) preserved the protein levels of BDNF and GDNF in the cerebellum (Figure 3A; ^##^ *p* < 0.01 vs. Ara-C-induced CA mice; *n* = 5).

Next, to investigate whether the hMSC treatment induced anti-neuroinflammatory effects in the cerebellum of the Ara-C-induced CA mice, we measured the protein levels of neurotoxic inflammatory molecules, such as IL-1β, TNF-α, and iNOS, in the cerebellum of the CA mice using Western blotting (Figure 3B). The Western blot results showed that the Ara-C-induced CA mice had significantly increased levels of IL-1β (2.704 ± 0.389), TNF-α (2.2 ± 0.15), and iNOS (2.31 ± 0.191) in the cerebellum compared to the nontreated intact mice (IL-1β: 1 ± 0.074; TNF-α: 1 ± 0.06; iNOS: 1 ± 0.061) (Figure 3B; *** *p* < 0.001 vs. nontreated intact mice). However, multiple hMSC treatments reduced the levels of inflammatory molecules (IL-1β: 1.386 ± 0.191; TNF-α: 1.011 ± 0.035; iNOS: 1.247 ± 0.09) in the cerebellum of the Ara-C-induced CA mice (Figure 3B; ^###^ *p* < 0.001 vs. Ara-C-induced CA mice; ^$^ *p* < 0.05 vs. single hMSC treatment in Ara-C-induced CA mice; *n* = 5). These results suggest that hMSC treatment increases the levels of neurotrophic factors and reduces neuroinflammation, thereby protecting cerebellar neurons in vivo.

## 4. Discussion

CA is a heterogeneous disease associated with a degeneration of the cerebellum and impaired coordination and balance [33]. Although there are several treatments available for CA, none of them can delay the progressive neurodegeneration caused by CA, and only symptom alleviation is possible [34]. Clinical and preclinical evidence has revealed an intriguing relationship between cerebellar inflammation and CA [19]. Stem cell therapies, particularly hMSC therapy, are promising for the treatment of CA due to their anti-inflammatory effects, capacity for regeneration via the release of neurotrophic factors, and ability to reduce immunogenicity [20,35]. Moreover, they exhibit no toxicity or tumorigenicity when transplanted into rodents or human patients [36,37,38,39] and reportedly attenuate motor dysfunction caused by gene mutation and drug exposure in vivo [16,35].

hMSCs act as factories for various bioactive factors; they not only produce several biochemical and molecular substances on their own [40] but also cause central nervous system remodeling by interacting with parenchymal tissue and triggering intrinsic healing processes [41]. Upon transplantation, they can move toward the site of injury [42]. They may be drawn to degenerating neurons via the chemokines they emit, making contact with such neurons and potentially directly supplying neurotrophic substances to suppress local inflammation and prevent degeneration [43]. MSCs derived from both animals and humans have been shown to secrete various well-known neurotrophic factors, leading to neurogenesis, cell differentiation, angiogenesis, reduction in free radical toxicity, inhibition of apoptosis, formation of glial scars, and neuronal and glial cell survival, thereby inducing neuroprotection and motor improvement in clinical trials with ataxia patients and preclinical ataxia models, such as SCA1-Tg, C57BL/6J-SCA2-Tg, and SCA3-MJD (Tg-ATXN3-Q69 MJD) [16,17,18,22,23,40,41,44,45,46].

We used an Ara-C model of ataxia, which exhibits clinical symptoms similar to those of CA, such as reduced motor performance, cerebellar neuronal damage, and cerebellar atrophy [24,25,26,27,47,48], and investigated whether single and/or multiple hMSC treatments can rescue the motor impairment and ataxia symptoms induced by Ara-C. Several mouse models mimicking the CA phenotype have been developed using chemicals, such as LPS [19] or ethanol [49]; mutant mice, such as Lurcher, Hootfoot, Weaver, and PCD mice; and genetically engineered mice, such as SCA1-Tg, C57BL/6J-SCA2-Tg, and SCA3-Tg MJD (Tg-ATXN3-Q69 MJD) [50]. The fundamental mechanism is characterized by a progressive functional and quantitative loss of brain cells, primarily Purkinje cells, which is the most frequent feature observed in ataxic patients and animal models with ataxic symptoms [51,52]. Furthermore, cortical neurons and Purkinje cells in the cerebellum are particularly vulnerable to damage caused by the production of excessive inflammatory mediators in overactivated glial cells. They serve as a crucial risk factor for CA progression as well as an attractive therapeutic target [53,54,55]. We revealed that, compared to single hMSC treatment, multiple hMSC treatments improved motor behavior and alleviated neuropathology in the Ara-C mouse model of CA. This may be due to the short lifetime of hMSCs after in vivo administration, which is a potential reason why some patients regress to the stage before MSC transplantation a few months after treatment [22]. A previous study on Tg-ATXN3-69Q (MJD) SCA3-Tg mice suggests that repeated mouse-born marrow-derived MSC administration can alleviate the SCA3-MJD phenotype via the preservation of Purkinje cells [46].

Regarding the therapeutic potential of hMSCs, compared to no treatment or single hMSC treatment, multiple hMSC treatments significantly improved the rotarod phenotypes and enhanced motor coordination, hindlimb clasping, and motor ability in the Ara-C-induced CA mice (Figure 1B–D). Further, immune blotting using calbindin and NeuN showed that multiple hMSC treatments significantly prevented the death of Purkinje and granule cells, both being the vital components of healthy motor coordination and sensory integration. (Figure 2A). Additionally, a lack of calbindin expression in Purkinje cells results in compromised motor coordination and processing of coordination-relevant visual information. Multiple hMSC treatments also induced an increase in cerebellar weight (Figure 2B). These results suggest that multiple hMSC transplantations are more effective in improving motor dysfunction and protecting cerebellar neurons from cell death than single hMSC transplantation (Figure 2).

Neurotrophic factors and inflammatory cytokines influence each other’s expression and function. Both in vitro and in vivo studies have shown that hMSCs migrate to the site of lesion and release trophic factors, such as BDNF, GDNF, IGF-1, and VEGF, as well as neuroregulatory factors, including SEM74 and cadherin-2, which are implicated in the survival of Purkinje neurons and attenuation of local inflammation [16]. Among these, BDNF and GDNF are some of the most active neurotrophins supporting the viability of existing neurons, thus promoting the growth and differentiation of new neurons [56,57,58,59,60,61,62]. According to a previous report, hMSCs can directly produce BDNF and GDNF in the injured brain. Furthermore, they may interact with host cells to activate an endogenous restorative mechanism to repair the injury [41]. Mice with targeted BDNF gene deletion display abnormal gait, increased granule cell death, and impaired Purkinje cell morphology, accompanied by a reduction in tropomyosin receptor kinase B activation, suggesting that BDNF directly targets both cell types in the cerebellum [59]. Moreover, BDNF also results in increased spine density in surviving Purkinje cells in vitro [60]. Notably, in the absence of BDNF, the secretome-induced axonal elongation effect is also lost [63]. Furthermore, according to patient samples with multiple system atrophy, GDNF is largely produced and localized in Purkinje cells of the cerebellum [64]. Additionally, GDNF induces dendritic thickening, spine development, and filopodial extensions in Purkinje cells [58]. A number of reports suggest that the loss of Purkinje cells is associated with inflammation, and the production of pro-inflammatory molecules is especially revealing in ataxia physiology as it contributes to cerebellar neuronal death in CA models [19,65]. Subsequently, increased levels of neurotrophic factors (BDNF and GDNF) are associated with reduced levels of various proinflammatory molecules, such as TNF-α, IL-1β, and iNOS (Figure 3). These results suggest that the paracrine effect induced by the hMSC administration plays a central role in inhibiting inflammatory responses, which is associated with the preserved weight of the cerebellum. Recently, we also demonstrated the therapeutic effects of hMSCs in an inflammatory CA model induced by LPS and found that hMSCs modulated microglial M2 polarization and inhibited apoptosis in the LPS-induced CA mouse model [20]. In the current study, we observed that single and multiple injections of hMSCs increased the levels of neurotrophic factors, but only multiple hMSC treatments reduced the levels of neuroinflammatory molecules. Hence, multiple hMSC treatments might be a promising therapeutic strategy for CA. As the precise mechanism of the interaction of hMSCs with host tissue to stimulate neurotrophins remains unclear, further studies are warranted. It would be interesting to compare the yield and outcome of the paracrine and anti-inflammatory effects induced by multiple MSC administrations with those of a specific secretome with a defined profile [16,63,66]. Additional study limitations include the evaluation of the potential risks of multiple transplantations and the quantity of grafted cells. Besides, this study is also limited to identifying the molecules that directly or indirectly interact with the host tissue to stimulate neurotrophins and the interactions with immune cells that mediate anti-inflammatory effects. Hence, further studies are needed to elucidate the mechanism by which hMSCs interact with Purkinje layers and mediate neuroprotection.

Altogether, in line with other reports, we suggest that the paracrine effect induced by hMSC administration plays a central role in Purkinje cell and granule cell survival, thus promoting cerebellar protection via an induction of neurotrophic levels, such as BDNF and GDNF. More importantly, it ameliorates the levels of pro-inflammatory molecules (TNF-α, IL-1β, and iNOS) and preserves motor impairment. Thus, we propose that hMSC administration, especially multiple hMSC administrations, might serve as a new potential therapeutic for ataxia pathology.

## 5. Conclusions

**Figure 4:**
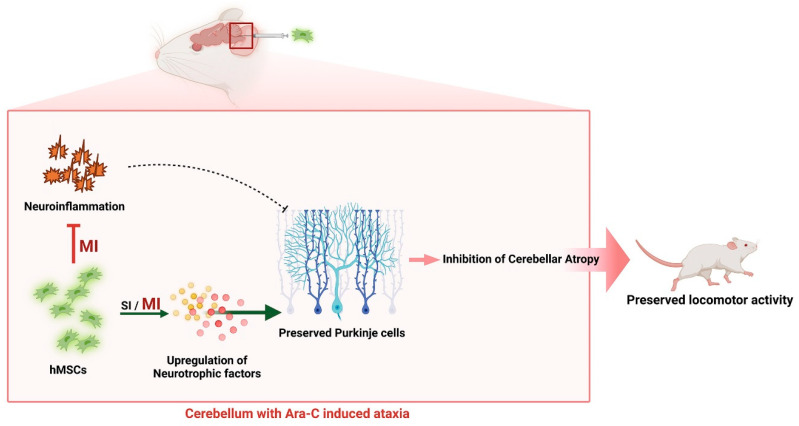
Schematic representation of the effects of hMSCs in the Ara-C-induced CA model. Multiple hMSC treatments significantly improve CA symptoms in the Ara-C-treated mice, and the beneficial effects are attributed to the inhibition of proinflammatory molecules and activation of neurotrophic factors, which prevent cerebellar weight loss and preserve locomotor activity. The figure was created using BioRender.com (Agreement number: HG24TP0M4C). Ara-C, cytosine arabinoside; hMSCs, human mesenchymal stem cells; SI, single injection; MI, multiple injection.

Increased levels of neurotrophic factors induced by hMSCs modulated immunology and promoted endogenous neuronal growth in the mouse model, thereby contributing to the therapeutic effect of hMSCs against Ara-C-induced CA pathophysiology (Figure 4). hMSC treatment, particularly multiple treatments, significantly improved CA symptoms in the Ara-C-treated mice. Furthermore, proinflammatory molecules were inhibited and neurotrophic factors were activated, which prevented cerebellar weight loss and preserved locomotion. These findings have considerable clinical significance for inducing hMSC administration in patients with CA.

## Data Availability

All data generated or analyzed during this study are included in this published article.
